# Tranexamic Acid-Associated Hyaluronic Acid Exhibits Enhanced Oxidative Stability: A Comparative Rheological Study

**DOI:** 10.3390/biom16030361

**Published:** 2026-02-28

**Authors:** Thierry Conrozier, Guillaume Darsy, Jérômine Mercier, Alexandre Guerry, Jérémy Patarin, Anne Lohse

**Affiliations:** 1 Département de Rhumatologie, Hôpital Nord Franche-Comté, 90400 Trevenans, France; anne.lohse@hnfc.fr; 2 Unité de Recherche Clinique, Hôpital Nord Franche-Comté, 90400 Trevenans, France; 3 Rheonova, Domaine Universitaire de Grenoble, 38400 Gières, France; darsy@rheonoca.fr (G.D.); patarin@rheonova.fr (J.P.); 4 Laboratoire de Rhumatologie Appliquée, 69001 Lyon, France; jeromine.mercier@labrha.com; 5 Biopolymer Heroes, 73370 Le Bourget du Lac, France; alexandre.guerry@bh-solution.com

**Keywords:** osteoarthritis, hyaluronic acid, tranexamic acid, viscosupplementation, rheology, oxidative stress

## Abstract

**Background:** The clinical performance of intra-articular hyaluronic acid (HA) is strongly dependent on its resistance to oxidative degradation within the inflamed osteoarthritic joint. Reactive oxygen species induce HA chain scission, leading to a loss of molecular entanglement and a shift from elastic-dominant to viscous-dominant behavior. Tranexamic acid (TXA), a lysine analogue with documented anti-inflammatory and anti-proteolytic properties, has been combined with HA with the hypothesis that it may limit oxidative-induced rheological degradation. **Objective:** This study aims to determine whether an HA–TXA formulation preserves viscoelastic integrity under oxidative stress and how its behavior compares with linear, hybrid, and cross-linked HA viscosupplements. **Methods:** Four HA-based formulations were evaluated using stress-controlled rotational rheometry compliant with ISO 3219 standards. Complex modulus (G*), complex viscosity (η*), and phase angle (tan δ) were measured within the linear viscoelastic domain. Oxidative challenge was induced with hydrogen peroxide (5.4% *v*/*v*), and time-dependent rheological changes were recorded over 30 min. Resistance to degradation was defined by relative variations in rheological parameters from baseline. **Results:** Baseline measurements revealed distinct viscoelastic profiles among the HA formulations. After oxidative exposure, the HA–TXA formulation showed a modest decrease in η* (−17.0%) and limited increase in tan δ (+4.0%), indicating preserved viscoelastic organization. Its stability exceeded that of hybrid (−40%; +12.6%) and linear HA (−53%; +25.6%) and approached that of cross-linked HA (−25.4%; +5.6%). The magnitude of microstructural alteration remained minimal despite chemical stress. **Conclusions:** The association of TXA with HA confers a marked protection against oxidative-induced viscoelastic degradation, preserving macromolecular network integrity and elastic behavior. These findings suggest that TXA modulates oxidative stress-related rheological failure of HA through mechanisms distinct from chemical cross-linking.

## 1. Introduction

Tranexamic acid (TXA) is a chemical compound that is structurally analogous to lysine. TXA is recognized for its high binding affinity toward the lysine-binding sites of plasminogen. Through this competitive mechanism, it obstructs the activation of plasminogen by its various activators, thereby inhibiting the generation of plasmin and the subsequent fibrinolytic cascade. In the context of joint disease, the plasminogen activator (PA) system is a critical regulator of matrix degradation [1]. An imbalance between PAs and their inhibitors (PAIs) leads to excessive plasmin production, which in turn triggers matrix metalloproteinases (MMPs), driving the destruction of the osteoarthritic cartilage. Several anti-osteoarthritic agents exert their effects by modulating the PA system. The receptor for urokinase-type PA (u-PA), expressed in the synovium of both osteoarthritis (OA) and rheumatoid arthritis (RA) patients, has been shown to be downregulated following treatment with hyaluronic acid [2,3]. Additionally, hydrocortisone’s anti-inflammatory and analgesic properties are partially attributed to the upregulation of PAI-1 expression and the concurrent suppression of tissue PA activity under both normal and inflammatory conditions [3]. TXA, even at submicromolar concentrations, attenuates plasminogen activation and significantly reduces plasmin production [4]. This antifibrinolytic effect underlies its widespread clinical use as a hemostatic agent in trauma management and major surgical procedures, including cardiac, orthopedic, and hepatic surgeries [5]. In vitro studies have suggested that plasmin contributes to OA pathogenesis through multiple mechanisms, including the degradation of proteoglycans and lubricin, as well as the induction of inflammatory and catabolic mediators [1]. Clinical evidence supports these findings: in patients undergoing knee arthroscopic arthrolysis, those receiving intra-articular administration of 500 mg TXA at the end of surgery demonstrated significantly lower pain scores and serum levels of pro-inflammatory markers, including interleukin-6 (IL-6) and C-reactive protein (CRP), one week postoperatively. Moreover, patients treated with TXA exhibited improved postoperative range of motion and higher Lysholm knee score [6]. Multiple studies support an anti-osteoarthritic role for TXA. In an OA animal model, Wang et al. demonstrated that pharmacological inhibition of plasmin attenuated OA progression, whereas plasmin injection exacerbated cartilage degeneration [1]. Similarly, Vignon et al. evaluated the effects of TXA in a rabbit model of OA induced by anterior cruciate ligament transection. Prophylactic intramuscular TXA administration three times per week for 3–6 months significantly suppressed stromelysin and collagenase activity, mitigating cartilage degradation [7]. Butler et al. further reported that in rabbits subjected to partial lateral meniscectomy and transection of the sesamoid and fibular collateral ligaments, both TXA and triamcinolone hexacetonide exhibited significant anti-osteoarthritic effects [8]. Additionally, TXA has been shown to reduce urinary collagen cross-link excretion in models of adjuvant-induced arthritis and rheumatoid arthritis, further supporting its disease-modifying potential [9].

It is well demonstrated that collagenases MMP-1 and MMP-13, by degrading type 2 collagen, play a major role in the degradation of the extracellular matrix (ECM) of hyaline cartilage [10]. Numerous inflammatory mediators, including interleukin (IL)-1β, IL-6, IL-17 and tumor necrosis factor α (TNF-α), are involved in the proteolytic degradation of articular cartilage. Their effects are mediated by binding to receptors including CD44, HA-mediated motility receptor (RHAMM) and Toll-like receptors (TLRs) [11,12]. IL-1β binds to receptors on articular cartilage chondrocytes and synovial cells and increases synthesis of matrix metalloproteinases (MMPs). IL-1β also stimulates chondrocyte production of ADAMTS (A Disintegrin And Metalloproteinase with Thrombospondin Motifs), a family of enzymes that degrade ECM aggrecans [13,14]. These findings have made MMPs the most attractive targets for the treatment of OA. However, non-proteolytic mechanisms of cartilage degradation may also contribute to the loss of extracellular matrix integrity [1]. The activation of fibrinolysis has been shown to promote OA development through multiple mechanisms, including the degradation of lubricin and cartilage proteoglycans and the induction of inflammatory and degradative mediators, suggesting that therapeutic targeting of the fibrinolysis pathway can prevent or slow development of OA. Indeed, in mice, genetic deficiency in the plasminogen gene *Plg* and pharmacological blockade of plasmin attenuate OA while genetic deficiency in plasminogen activator inhibitor (PAI) accelerates OA [1]. Finally, IL-1β also increases nitric oxide (NO) synthesis and NADPH oxidase 4 and decreases the expression of superoxide dismutase and glutathione peroxidase, leading to an acceleration of the damaging effects of oxygen radicals on the extracellular matrix [15].

Intra-articular (IA) injections of hyaluronic acid (HA) have been used worldwide to treat OA of the knee for over 30 years [16]. The use of HA-IA to treat OA is justified by the fact that HA, beyond its viscoelastic properties, exerts anti-nociceptive and anti-inflammatory actions [17] and has demonstrated disease-modifying effects in vitro and in vivo, beneficial for the preservation of the extracellular matrix. The current literature confirms that HA is not just a medical device used for joint lubrication, but a biologically active molecule that can improve articular cartilage physiology [17,18]. However, the percentage of patients with knee osteoarthritis who are satisfied with VS does not exceed 70% to 80% at 6 months and is around 50% at 1 year [19], but varies greatly depending on the severity of the osteoarthritis, the characteristics and lifestyle of the patients, and the products used [20]. Optimizing the clinical efficacy of IA-HA is an exciting challenge for manufacturers who are developing new formulations by adding various active molecules (i.e., antioxidants, corticosteroids, NSAIDs, gold microparticules, bisphosphonates, etc...) [21,22,23,24,25] in order to increase or prolong the effect of HA.

In light of the potential benefits of TXA in OA and its widespread use in orthopedic surgery, with excellent intra-articular tolerance being demonstrated, the logical step was to consider a combination with HA. This combination is hypothesized to protect the latter from the deleterious effects of enzymes produced by synovial inflammation, as well as to utilize the possible chondroprotective effect of TXA. This is how an innovative viscosupplement combining high-molecular-weight linear HA (2.2 MDa) at a concentration of 22 mg/mL and TXA at a concentration of 15 mg/mL has been developed (LABRHA.SAS laboratory, Lyon, France).

However, before any clinical application, it was essential to confirm that the addition of TXA does not substantially alter the three-dimensional structure of HA, and consequently that the HA-TXA combination possesses the required viscoelastic properties for a viscosupplement.

Furthermore, it must be demonstrated that the HA-TXA combination is resistant to oxidative stress to a similar degree to HA, since ROS-related degradation is the main cause of HA short half-life in OA joints.

Rheological testing is an inexpensive and valuable way to evaluate the microstructure of a viscosupplement because any change in rheological behavior indicates a change in the gel’s three-dimensional network. The objective of this study was to compare, in vitro, the rheological properties as well as the resistance to the ROS-mediated degradation of the HA-TXA combination with that of three commercially available viscosupplements differing in molecular structure (linear or cross-linked) and HA concentration (2% to 3.2%). This comparison was conducted by analyzing the rheological properties before and after exposure to hydrogen peroxide (H_2_O_2_), a well-established and validated method for degrading polysaccharides via a radical mechanism [26].

Nevertheless, it should be emphasized that this in vitro model is limited to observing rheological changes directly induced by H_2_O_2_ and does not permit the study of all phenomena occurring in vivo. This is particularly pertinent with regard to the effects of TXA on the inflammatory cascade induced by PA inhibition.

## 2. Materials and Methods

### 2.1. Rheological Equipment

To characterize the mechanical behavior of the HA-TXA blend, we compared it against three distinct categories of viscosupplements: a linear HA, a hybrid complex, and a cross-linked gel. The main physicochemical characteristics of these products are summarized in Table 1. All rheological evaluations were performed using a DHR3 stress-imposed rheometer (TA Instruments) utilizing a 25 mm diameter cross-hatched plate–plate system to prevent slippage, following the technical guidelines of ISO 3219. Experimental procedures complied with ISO 3219-1 and ISO 3219-2 [27,28] recommendations for rotational rheometry. Temperature control was provided by a Peltier system integrated into the lower plate. Prior to testing, all samples were stored in their original packaging at ambient temperature (<25 °C) and protected from light. During measurements, the temperature of the lower plate was maintained at 25 °C with an accuracy of ±0.5 °C. The zero-gap position of the geometry was determined according to the manufacturer’s protocol and re-established whenever the geometry was removed, notably for cleaning. Before each experimental session, routine instrument calibrations were performed, including geometry inertia, air-bearing friction and magnetic bearing mapping, following the procedures supplied by the rheometer manufacturer.

### 2.2. Sample Preparation and Loading

Immediately before analysis, samples were equilibrated at room temperature (23 °C) and loaded directly from their syringes onto the lower plate without the use of needles. The upper plate was lowered to an initial trimming gap of 1050 µm, excess material was carefully removed using a spatula, and the final measurement gap was then adjusted to 1000 µm.

### 2.3. Oscillatory Strain Sweep Measurements

Oscillatory strain sweep tests were performed to characterize the viscoelastic behavior of the samples under resting conditions and to assess their gel-like nature. These measurements followed widely accepted and standardized rheological protocols. The applied strain ranged from 0.001% to 3000% at a fixed oscillation frequency of 1 Hz, without any pre-shear step. For each product and each experimental condition, at least two reproducible measurements were obtained. The linear viscoelastic domain (LVED) was identified, and rheological parameters were extracted by averaging data points within this region. The complex modulus (G*, expressed in Pa) and the phase angle tangent (tan δ) were used as primary descriptors of material behavior. Tan δ, defined as the ratio between the loss modulus (G″) and the storage modulus (G′), provides information on the relative contributions of viscous and elastic components. Values below unity within the LVED were considered indicative of gel-like behavior. The complex modulus G* reflects the overall mechanical resistance of the material under oscillatory deformation and results from the combined elastic and viscous responses. Because viscosity is intrinsically a flow-dependent property and strain sweep tests are quasi-static, modulus-based parameters (G* and tan δ), together with the complex viscosity (η*), were deemed the most relevant descriptors in this experimental context. For viscoelastic materials such as hyaluronic acid formulations, complex rheological parameters are commonly used to characterize their response to oscillatory stress. The complex viscosity η* describes resistance to flow and is related to the complex modulus by the equation η* = G*/(2πf), where f is the oscillation frequency, although the two parameters represent distinct physical concepts. For each parameter, mean values and relative standard deviations were calculated. Samples were tested in a randomized sequence, and the operator was blinded to product identity until completion of the rheological analyses, after which compositions were disclosed for interpretation. Each formulation was measured twice. The coefficients of variation were 3.35% for G*, 2.8% for η*, and 3.82% for tan δ, indicating good measurement consistency. The overall standard errors of the mean (95% confidence interval) were 2.53 Pa [208.7–218.6], 0.337 Pa·s [33.35–34.67], and 0.013 [0.94–0.99] for G*, η*, and tan δ, respectively. Values reported in the Section 3 represent the mean of duplicate measurements with their corresponding standard deviations. Owing to the limited sample size, the data are presented descriptively, as formal statistical comparisons would not provide meaningful interpretation.

### 2.4. Oxidative Stress Protocol

The susceptibility of the four hyaluronic acid-based viscosupplements to oxidative breakdown was assessed under hydrogen peroxide-induced stress conditions. The oxidative insult was generated with H_2_O_2_ (30%, Sigma-Aldrich) adjusted to a final concentration of 5.4% (*v*/*v*). For each product, 1 mL of formulation was placed on the lower rheometer plate, after which 0.1 mL of hydrogen peroxide was introduced, ensuring a fixed volume ratio of 10:1. Because of the restricted sample quantity, the preparation was manually mixed for 10 s using a spatula with circular movements at roughly one revolution per second, prior to installation of the upper plate according to the previously described procedure. The time elapsed between H_2_O_2_ addition and acquisition of the first data point was monitored using a stopwatch and averaged one minute (±5 s), mainly depending on thermal equilibration of the system. The measured delay was used to correct the experimental start time for each test. Oxidative stress experiments were conducted over a total duration of 30 min. The evolution of rheological properties under oxidative conditions was continuously monitored using oscillatory time sweep measurements performed at a frequency of 1 Hz and a strain amplitude of 1%, selected to remain within the LVED. Changes in G*, tan δ and η* were recorded in real time throughout the exposure period.

## 3. Results

The detailed results of G*, tan δ and η* for the various viscosupplements studied are summarized in Table 2.

### 3.1. Strain Sweeps

At baseline, before oxidative stress, the different HA formulations exhibited distinct rheological behaviors. As shown in Table 2, the complex modulus (G*) ranged from 31.81 to 401.35 Pa. A higher G* was associated either with a cross-linked structure or a high molecular weight, but not with HA concentration. Notably, the lowest G* value was observed in the hybrid HA formulation, despite its highest concentration (3.2%). Regarding tan δ, the cross-linked HA displayed a gel-like behavior with a value of 0.2, indicating a predominant contribution of G’, the storage modulus, which represents the elastic (solid-like) component. For linear HA formulations, a gel-like state was observed in the highest molecular weight HAs (HA and HA-TXA). However, these gels exhibited a lower solid-like character compared to cross-linked HAs, with tan δ values of 0.52 and 0.75, respectively.

### 3.2. Mechanical Stress

We first assessed the mechanical impact of mixing HA with hydrogen peroxide on rheological properties. During oxidation tests, the more structurally consistent the sample, the more challenging the mixing process, requiring increased mechanical agitation. The effect of this mixing can be evaluated by considering thixotropy—specifically, the temporal recovery of rheological properties following mechanical stress. This was quantified by analyzing variations in G*, tan δ, and η* between the LVED in the absence of oxidative stress and their respective maximum (or minimum, for tan δ) values recorded during oxidation tests.

### 3.3. Oxidative Stress

**Figure 1:**
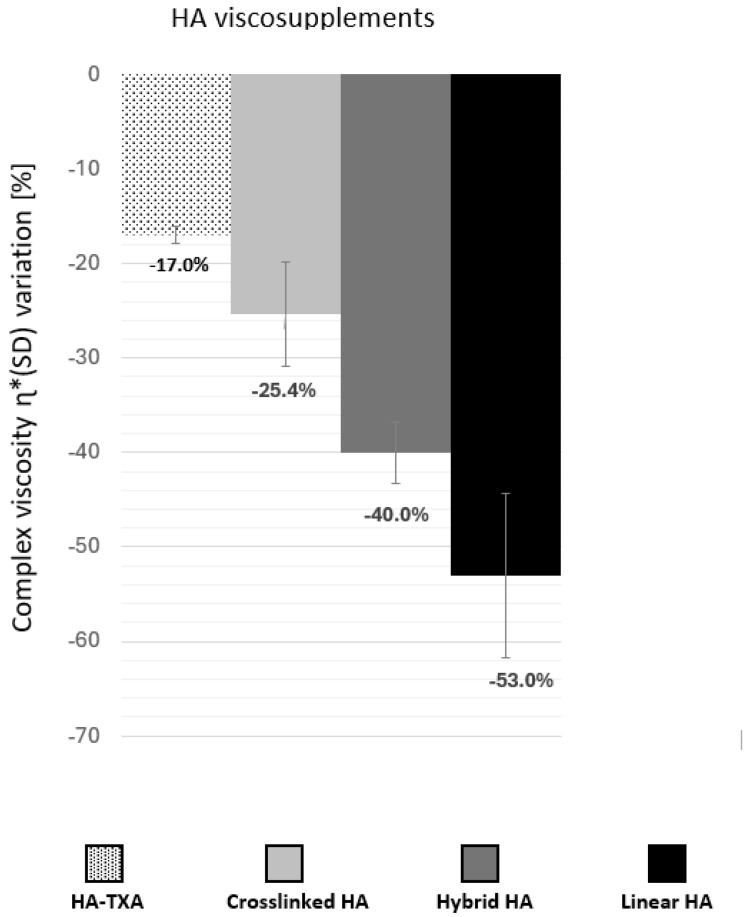
Percentage of decrease in the complex viscosity Δη* (%) between the values in the linear viscoelastic domain and the end of oxidation tests according to the structure of various viscosupplements. HA-TXA vs. Cross-linked HA (*p* = 0.67); HA-TXA vs. Hybrid HA (*p* = 0.03); HA-TXA vs. linear HA (*p* = 0.01).

**Figure 2:**
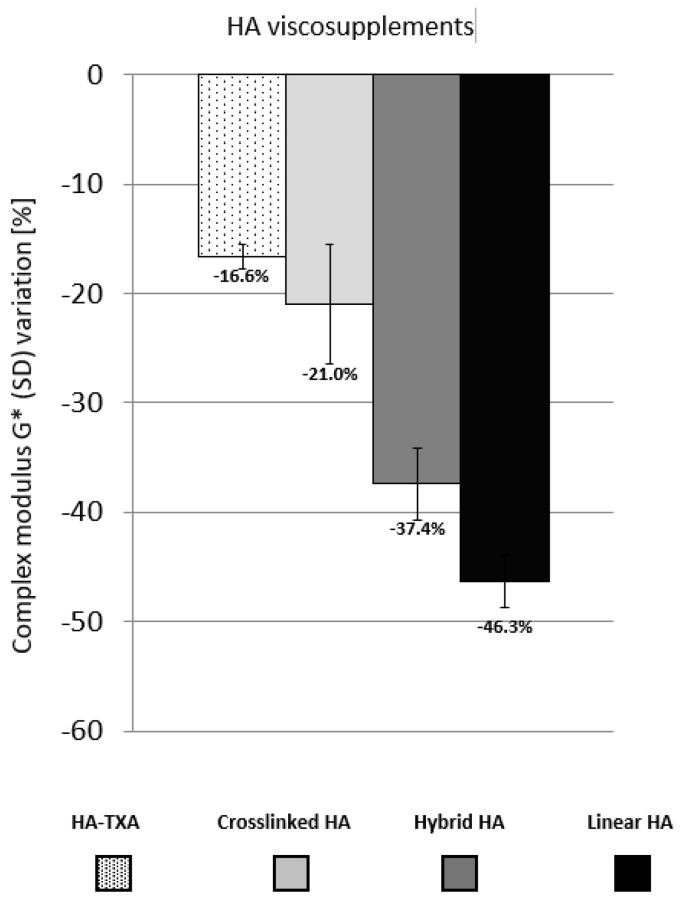
Percentage of variation in the complex modulus G* between the values in the linear viscoelastic domain and the end of oxidation tests for different viscosupplements. HA-TXA vs. Cross-linked HA (*p* = 0.87); HA-TXA vs. Hybrid HA (*p* = 0.04); HA-TXA vs. linear HA (*p* = 0.02).

To evaluate the impact of the oxidative stress, by taking into account the mixing process, the percentage of variation in G* or tan δ was evaluated. It was calculated as the relative difference between the values in the LVED and the final values of the oxidizing tests. Following oxidative stress, η* decreased by 1.25%, 5.9% %, 6.6%, and 33.1% for cross-linked HA, HA-TXA, hybrid HA and linear HA, respectively. Combining mechanical and oxidative stresses, the HA-TXA combination (Δη* ± SD = −17.0 ± 0.9%) and cross-linked HA (Δη* ± SD = −25.4 ± 5.5%) showed superior resistance to degradation compared to hybrid HA (Δη* ± SD = −40 ± 3.3%) and linear HA (Δη* ± SD = −53 ± 8.7%) (Figure 1). Not surprisingly, G* variations between LVED and the end of oxidative stress presented a similar profile (Figure 2) indicating a greater loss of consistency of linear and hybrid viscosupplements.

**Figure 3:**
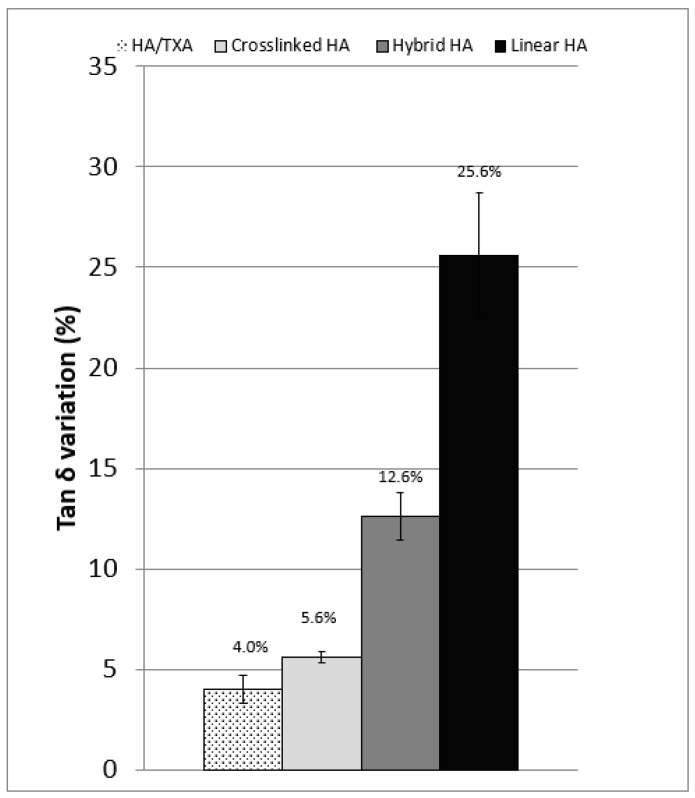
Percentage of variation in tan δ between the values in the linear viscoelastic domain and the end of oxidation tests for different viscosupplements. HA-TXA vs. Cross-linked HA (*p* = 0.81); HA-TXA vs. Hybrid HA (*p* = 0.01); HA-TXA vs. linear HA (*p* = 0.007).

The microstructural signature was assessed through variations in tan δ. Figure 3 shows that both the cross-linked product and the HA-TXA combination underwent mild microstructural modifications, resulting in a slightly more pronounced viscous liquid behavior, reflected by a moderate increase in tan δ. In contrast, for the linear and hybrid viscosupplements, the changes were more substantial, leading to a significantly more pronounced viscous liquid character. Indeed, tan δ reflects the balance between viscous and elastic behavior and is commonly considered a microstructural indicator of polymer network integrity. An increase in tan δ following oxidative stress denotes a shift toward more liquid-like behavior, consistent with reactive oxygen species-mediated fragmentation of HA chains and weakening of interchain interactions. In the present study, the large Δtan δ observed for linear and hybrid formulations suggests substantial disruption of the three-dimensional network, whereas the limited increase seen with cross-linked HA and the HA–TXA combination indicates better preservation of the viscoelastic balance despite chemical degradation. When interpreted together with the smaller decreases in G* and η*, these findings support the maintenance of supramolecular organization under stress. Although not a direct surrogate for clinical efficacy, preservation of a low tan δ is consistent with retention of gel-like behavior that may favor shock absorption, load distribution, and potentially longer intra-articular residence time.

## 4. Discussion

Our study provides an imperfect yet informative representation of the fate of HA in OA joints following injection. As soon as it is injected into the joint cavity, HA is exposed to both mechanical stress, due to joint movement, and oxidative stress, resulting from the presence of reactive oxygen species. In a previous study using the same oxidative stress model [26], we had already highlighted significant differences in degradation resistance among viscosupplements. We demonstrated that cross-linked products exhibited substantially greater resistance to mechanical and oxidative stresses compared to linear HAs, thereby explaining their significantly longer half-lives. In the present study, the cross-linked viscosupplement undergoes markedly less degradation than the linear formulations, including both the high-molecular-weight HA and the hybrid formulation composed of intermediate- and low-molecular-weight HAs. Cross-linking refers to the formation of covalent bridges between individual linear strands of hyaluronic acid (HA) through chemical or physical processes. This network organization generates a three-dimensional hydrogel that significantly improves the rheological behavior of the material while increasing its structural integrity and persistence compared to native HA. Experimental data have demonstrated that this network formation significantly prolongs the intra-articular longevity of HA and improves its viscoelastic performance. As a result, cross-linked formulations can remain in situ for periods of up to four weeks, which is up to ten times longer than linear HA (ref.). Darsy et al. [26] have thus demonstrated major differences between cross-linked and linear products, with the latter being 4.5 times more degraded than the former under the same oxidative stress. Our findings, as with the previous ones, clearly demonstrated that cross-linking, and consequently the formation of a gel-like structure, confers a protective advantage by mitigating the detrimental effects of both oxidative and mechanical stress on the macromolecular integrity of HA [29,30]. In contrast, the molecular weight (MW) of HA appears to play a less significant role in resistance to degradation. Although, MW was not directly measured in our study, it was inferred from complex viscosity values, which indirectly reflect MW. This is supported by the higher resistance to H_2_O_2_-induced degradation of hybrid HA (ΔG* = −34.4%) compared to linear HA (ΔG* = −46.3%), despite the latter having a substantially higher MW and viscosity.

The unexpected finding of our study lies in the high resistance to degradation of the HA-TXA combination, despite being composed of linear HA with an MW similar to that of the linear HA used as a comparator. We have no clear explanation for the increased stability of the HA-TXA combination, as rheology only allows us to observe the facts but not to explain them. One could hypothesize a structuring effect of TXA, which could induce interaction between HA chains, thereby improving resistance to hydrogen peroxide-mediated degradation. However, if this were the case, prior to oxidative stress, the tan δ, which indicates whether the material is gel-like or not, should have been lower than that of the other linear HA, whereas it is of the same order of magnitude. As demonstrated by Chytil et al. [31], adding lysine decreases HA viscosity. This is attributed to the breakdown of interchain interactions, particularly hydrogen bonds, and the formation of point interactions between the lysine groups and the glucuronic acid residues of HA. Since TXA is an analogue of lysine, as it shares polar functional groups (−COOH and −NH_2_) that are capable of interacting with HA, its behavior with regard to HA should be the same. Based on Chytil’s findings, it is reasonable to assume that, during the initial phase of our study, the ionic and hydrogen bonds formed between TXA and HA disrupted the supramolecular architecture of the HA network. This effect would be a consequence of the rigid and relatively hydrophobic nature of the cyclohexane structure of TXA and could explain the initial decrease in the viscosity of AH-TXA compared to linear AH. Conversely, to explain the greater resistance to oxidation of the HA-TXA combination, it can be imagined that H_2_O_2_, by causing fragmentation of the HA chains, makes the functional groups more accessible and promotes TXA-HA interactions through various pathways. The NH_3_^+^ groups of TXA could thus form ionic and hydrogen bonds with the COO^−^ groups of glucuronic acid residues, and the weakly polar cyclohexane ring of TXA could enter into weak hydrophobic interactions with the N-acetylglucosamine units of HA. It can also be hypothesized that TXA-TXA hydrophobic interactions may have formed via the cyclohexane rings. This non-covalent mesh could contribute to the formation of a supramolecular network, limiting the loss of mechanical properties despite the base polymer’s chemical degradation. Although HA is degraded (fragmentation and a decrease in molar mass occur), this network of physical interactions results in a higher η* and G* modulus than linear HA exposed to the same oxidative stress alone. Finally, the potential intrinsic antioxidant activity of TXA should also be considered, as this could also contribute to its protective capacity against HA. Tranexamic acid (TXA), beyond its well-established antifibrinolytic activity, has also been reported to exert antioxidant effects in experimental models. In naturally aging mice, TXA administration was shown to reduce circulating reactive oxygen species and improve survival, suggesting systemic antioxidative action [32]. In a rat model of ischemia–reperfusion-induced lung injury, TXA decreased oxidative stress biomarkers such as malondialdehyde and ischemia-modified albumin, thereby attenuating tissue damage [33]. At the cellular level, TXA has been demonstrated to suppress the release of mitochondrial DNA, protect endothelial monolayers, and enhance oxidative phosphorylation, mechanisms consistent with a reduction in oxidative stress and mitochondrial dysfunction [34]. Together, these findings indicate that TXA exerts a moderate but biologically relevant antioxidant effect, which may complement its anti-inflammatory properties, although this remains ancillary to its primary clinical role as an antifibrinolytic agent.

Our work does not allow us to draw any formal conclusions regarding the mechanisms by which TXA protects AH from oxidative stress. The rheology demonstrates the facts but does not provide an explanation for the reason for this phenomenon. Consequently, there is an absence of evidence to support our hypothesis. Conducting material characterization experiments, such as nuclear magnetic resonance, Fourier transform infrared spectroscopy, dynamic light scattering, scanning or transmission electron microscopy, X-ray diffraction, or mass spectrometry, would allow verification of whether TXA forms stable complexes with HA. Furthermore, molecular docking and molecular dynamics simulations could be utilized to provide theoretical substantiation for the proposed mechanism.

In our study, HA concentration (ranging from 20 to 32 mg/mL) does not appear to confer protection against ROS-mediated degradation. Indeed, the most concentrated viscosupplement, hybrid HA (HA concentration: 3.2%), was far more susceptible to oxidative degradation than the HA-TXA combination and cross-linked HA.

One of the major strengths of this study lies in the implementation of standardized rheological protocols widely recognized by the industry. Measurements were performed using a stress-controlled rheometer in compliance with ISO 3219, thereby ensuring both reproducibility and methodological consistency. In addition, to minimize potential bias during product comparison, samples were analyzed in a randomized order, and investigators were blinded to the identity of the formulations, preventing any procedural bias. One limitation of our study is that we cannot extrapolate our findings in favor of the superiority of HA-TXA over HA to all viscosupplements available on the market. Nevertheless, our recently published study [26], using the same experimental model, showed similar results with other linear (ΔG* ranging from −29.6% to −44.9%) and cross-linked (ΔG* ranging from −10.1% to −25.4%) viscosupplements. Among other limitations of our work, the limited sample size and the absence of statistical hypothesis testing reduce the reliability of the reported differences. Furthermore, the study uses a 30-min oxidative stress protocol. It is unknown how the formulations would perform over longer periods or under cyclic loading mimicking joint activity over several days/weeks. Finally, while this rheological model has been validated, it only provides an approximate representation of the processes occurring in the joint. Therefore, great caution must be exercised when extrapolating these results to physiological conditions.

Our findings suggest that, beyond its intrinsic anti-arthritic effect through plasminogen activator inhibition [1], TXA may prolong the intra-articular residence time of HA by shielding it from ROS-induced degradation. By extending HA’s contact time with target tissues, particularly synovial nociceptors, TXA could potentiate HA’s analgesic effects. It is worth noting that the result of this in vitro study does not necessarily imply improved biological outcomes (e.g., analgesia, anti-inflammation). It is essential to distinguish between physical stability and clinical performance, and to avoid suggesting the therapeutic superiority of the HA-TXA combination unless this is supported by functional or in vivo data. However, the clinical superiority of the HA-TXA combination over HA alone has been suggested in an animal model. The anti-nociceptive and chondroprotective effects of the HA-TXA combination were evaluated by Baugé et al. in a murine model of osteoarthritis (OA) [35]. Knee OA was induced in mice via intra-articular (IA) injection of monosodium iodoacetate (MIA). The animals were treated with IA injections of saline, HA, or HA-TXA, and tactile sensitivity was assessed using von Frey filaments. The pain threshold was significantly higher in HA-TXA-treated mice compared to those receiving HA or saline throughout the 28-day follow-up period. On day 56, the mice were euthanized for histological knee assessment using the OARSI score, which was significantly lower in HA-TXA- and HA-treated mice compared to the saline-treated group. This study suggests that the addition of TXA to HA enhances its analgesic effect without compromising its ability to slow the progression of osteoarthritis. Although the mechanism by which the HA-TXA combination works is unclear, it is reasonable to assume that this is at least partly due to TXA’s well-documented anti-inflammatory properties [36,37,38]. A randomized controlled trial comparing HA-TXA and HA alone over a period of 12 months is currently underway in 252 patients with knee osteoarthritis (clinicaltrials.gov NCT05978180). If the ongoing clinical study confirms the results of the animal study—that is, that HA-TXA is more effective than HA at relieving pain for 12 months—a new opportunity will arise for prescribers.

## 5. Conclusions

Our findings confirm that cross-linking—and the resulting gel-like structure—provides a protective advantage by preserving the macromolecular integrity of HA against both oxidative and mechanical stress. More intriguingly, they reveal that the addition of TXA, even at low concentrations, safeguards HA from ROS-induced degradation. While preceding research focused on the biological and anti-inflammatory pathways of HA-TXA in a murine model, the present study offers a distinct biophysical perspective. In this study, we demonstrate that the addition of TXA does not merely act as a pharmacological agent but fundamentally alters the rheological resilience of the hyaluronan network. This work underscores the oxidative stability exhibited by the HA-TXA combination, which rivals, and, in certain parameters, exceeds that of chemically cross-linked gels. The underlying mechanism of this stability remains unelucidated due to the limitations of the design of our study. Other studies, particularly in vivo studies, are already underway to assess whether or not the HA-TXA combination prolongs the effect of viscosupplementation.

## Figures and Tables

**Table 1:** Characteristics of the studied hyaluronic acid viscosupplements.

Branded Name	Manufacturer	Structure	HA Concentration	HA MW
ARTHRUM VISC 75^®^	LCA pharmaceutical Chartres, France	Linear HA	2.5%	2.4 MDa
HYAJOINT PLUS^®^	SciViscion Biotech Inc. Kaohsiung, Taiwan	Cross-linked HA	2%	NA
PANDORA^®^	LABRHA. Lyon, France	Linear HA-TXA	2.2%	2.2 MDa
SINOVIAL HL^®^	IBSA Farmaceutici Lodi, Italia	Hybrid linear HA	3.2%	1:1 ratio of 0.08–0.1 MDa 1.4–2.1 MDA

**Table 2:** Average values of complex modulus G*, tangent δ and complex viscosity η*, in the linear domain obtained with the strain sweep and time sweep (before and after oxidation) according to the viscosupplement.

		Oscillations: Strain Sweep			Oscillations: Time Sweep					
		Linear Domain			Initial Values Before Oxidation			Final Values After Oxidation		
		G*	Tan δ	η*	G*	Tan δ	η*	G*	Tan δ	η*
Sample	Value	Pa	-	Pa.s	Pa	-	Pa.s	Pa	-	Pa.s
HA-TXA	Mean	253.20	0.756	40.32	224.62	0.73	35.75	211.29	0.76	33.63
	SD	9.97	0.02	1.65	8.20	0.02	1.81	6.50	0.03	0.99
Crosslinked HA	Mean	169.19	0.22	26.93	134.93	0.26	21.47	133.25	0.24	21.21
	SD	8.80	0.008	1.39	6.60	0.10	0.98	10.0	0.008	1.54
Hybrid HA	Mean	31.81	2.36	5.05	21.28	2.61	3.39	19.87	2.94	3.16
	SD	1.40	0.008	0.23	1.33	0.009	0.27	1.42	0.12	0.29
Linear HA	Mean	401.35	0.52	63.81	322.29	0.55	51.29	215.57	0.73	34.31
	SD	4.80	0.026	0.65	6.01	0.047	0.66	5.13	0.033	0.89

## Data Availability

The original contributions presented in this study are included in the article. Further inquiries can be requested at the following address: pandora@labrha.com.

## References

[1] Wang Q., Shao G., Zhao X., Wong H.H., Chin K., Zhao M., Bai A., Bloom M.S., Love Z.Z., Chu C.R. (2024). Dysregulated fibrinolysis and plasmin activation promote the pathogenesis of osteoarthritis. JCI Insight.

[2] Masuko K., Murata M., Yudoh K., Kato T., Nakamura H. (2009). Anti-inflammatory effects of hyaluronan in arthritis therapy: Not just for viscosity. Int. J. Gen. Med..

[3] Nonaka T., Kikuchi H., Ikeda T., Okamoto Y., Hamanishi C., Tanaka S. (2000). Hyaluronic acid inhibits the expression of u-PA, PAI-1, and u-PAR in human synovial fibroblasts of osteoarthritis and rheumatoid arthritis. J. Rheumatol..

[4] Wu G., Mazzitelli B.A., Quek A.J., Veldman M., Conroy P.J., Caradoc-Davies T.T., Ooms L.M., Tuck K.L., Schoenecker J.G., Whisstock J.C. (2019). Tranexamic acid is an active site inhibitor of urokinase plasminogen activator. Blood Adv..

[5] Pai B.H.P., Patel S., Lai Y.H. (2023). Updated Clinical Review: Perioperative Use of Tranexamic Acid in Orthopedics and Other Surgeries. Adv. Anesth..

[6] Li J., You M., Yao L., Fu W., Li Q., Chen G., Tang X., Li J., Xiong Y. (2023). Topical administration of tranexamic acid reduces postoperative blood loss and inflammatory response in knee arthroscopic arthrolysis: A retrospective comparative study. BMC Musculoskelet. Disord..

[7] Vignon E., Mathieu P., Bejui J., Descotes J., Hartmann D., Patricot L.M., Richard M. (1991). Study of an inhibitor of plasminogen activator (tranexamic acid) in the treatment of experimental osteoarthritis. J. Rheumatol. Suppl..

[8] Butler M., Colombo C., Hickman L., O’Byrne E., Steele R., Steinetz B., Quintavalla J., Yokoyama N. (1983). A new model of osteoarthritis in rabbits. III. Evaluation of anti-osteoarthritic effects of selected drugs administered intraarticularly. Arthritis Rheum..

[9] Ronday H.K., Te Koppele J.M., Greenwald R.A., Moak S.A., De Roos J.A., Dijkmans B.A., Breedveld F.C., Verheijen J.H. (1998). Tranexamic acid, an inhibitor of plasminogen activation, reduces urinary collagen cross-link excretion in both experimental and rheumatoid arthritis. Br. J. Rheumatol..

[10] Iannone F., Lapadula G. (2003). The pathophysiology of osteoarthritis. Aging Clin. Exp. Res..

[11] Fernandes J.C., Martel-Pelletier J., Pelletier J.P. (2002). The role of cytokines in osteoarthritis pathophysiology. Biorheology.

[12] Pérez-García S., Carrión M., Gutiérrez-Cañas I., Villanueva-Romero R., Castro D., Martínez C., González-Álvaro I., Blanco F.J., Juarranz Y., Gomariz R.P. (2019). Profile of Matrix-Remodeling Proteinases in Osteoarthritis: Impact of Fibronectin. Cells.

[13] Dunn S., Kolomytkin O.V., Waddell D.D., Marino A.A. (2009). Hyaluronan-binding receptors: Possible involvement in osteoarthritis. Mod. Rheumatol..

[14] Durigova M., Troeberg L., Nagase H., Roughley P.J., Mort J.S. (2011). Involvement of ADAMTS5 and hyaluronidase in aggrecan degradation and release from OSM-stimulated cartilage. Eur. Cell Mater..

[15] Drevet S., Gavazzi G., Grange L., Dupuy C., Lardy B. (2018). Reactive oxygen species and NADPH oxidase 4 involvement in osteoarthritis. Exp. Gerontol..

[16] Balazs E.A., Denlinger J.L. (1993). Viscosupplementation: A new concept in the treatment of osteoarthritis. J. Rheumatol. Suppl..

[17] Nicholls M.A., Fierlinger A., Niazi F., Bhandari M. (2017). The Disease-Modifying Effects of Hyaluronan in the Osteoarthritic Disease State. Clin. Med. Insights Arthritis Musculoskelet. Disord..

[18] Henrotin Y., Chevalier X., Raman R., Richette P., Montfort J., Jerosch J., Baron D., Bard H., Carrillon Y., Migliore A. (2020). EUROVISCO Guidelines for the Design and Conduct of Clinical Trials Assessing the Disease-Modifying Effect of Knee Viscosupplementation. Cartilage.

[19] Conrozier T., Mathieu P., Schott A.M., Laurent I., Hajri T., Crozes P., Grand P., Laurent H., Marchand F., Meignan F. (2003). Factors predicting long-term efficacy of Hylan GF-20 viscosupplementation in knee osteoarthritis. Joint Bone Spine.

[20] Conrozier T., Raman R., Diraçoglu D., Montfort J., Bard H., Baron D., Goncalves B., Richette P., Migliore A., Chevalier X. (2025). EUROVISCO Consensus Guidelines for the Use of Hyaluronic Acid Viscosupplementation in Knee Osteoarthritis Based on Patient Characteristics. Cartilage.

[21] Conrozier T. (2018). Is the Addition of a Polyol to Hyaluronic Acid a Significant Advance in the Treatment of Osteoarthritis?. Curr. Rheumatol. Rev..

[22] Hangody L., Szody R., Lukasik P., Zgadzaj W., Lénárt E., Dokoupilova E., Bichovsk D., Berta A., Vasarhelyi G., Ficzere A. (2018). Intraarticular Injection of a Cross-Linked Sodium Hyaluronate Combined with Triamcinolone Hexacetonide (Cingal) to Provide Symptomatic Relief of Osteoarthritis of the Knee: A Randomized, Double-Blind, Placebo-Controlled Multicenter Clinical Trial. Cartilage.

[23] Badawi A.A., El-Laithy H.M., Nesseem D.I., El-Husseney S.S. (2013). Pharmaceutical and medical aspects of hyaluronic acid-ketorolac combination therapy in osteoarthritis treatment: Radiographic imaging and bone mineral density. J. Drug Target..

[24] Rasmussen S., Petersen K.K., Aboo C., Andersen J.S., Skjoldemose E., Jørgensen N.K., Stensballe A., Arendt-Nielsen L. (2024). Intra-articular injection of gold micro-particles with hyaluronic acid for painful knee osteoarthritis. BMC Musculoskelet. Disord..

[25] Scanu A., Luisetto R., Pavan M., Guarise C., Beninatto R., Giraudo C., Galuppini F., Lazzarin V., Guzzardo V., Pennelli G. (2023). Effect of intra-articular injection of a hyaluronic acid-alendronate combination on post-traumatic osteoarthritis induced by destabilization of the medial meniscus in rats. Sci. Rep..

[26] Darsy G., Patarin J., Conrozier T. (2023). Large Variations in Resistance to Degradation between Hyaluronic Acid Viscosupplements: A Comparative Rheological Study. Cartilage.

[27] (2021). Rheology—Part 1: Vocabulary and Symbols for Rotational and Oscillatory Rheometry.

[28] (2021). Rheology—Part 2: General principles of rotational and oscillatory rheometry.

[29] Bayer I.S. (2020). Hyaluronic Acid and Controlled Release: A Review. Molecules.

[30] Luo Z., Wang Y., Xu Y., Wang J., Yu Y. (2023). Modification and crosslinking strategies for hyaluronic acid-based hydrogel biomaterials. Smart Med..

[31] Chytil M., Trojan M., Kovalenko A. (2016). Study on mutual interactions and electronic structures of hyaluronan with Lysine, 6-Aminocaproic acid and Arginine. Carbohydr. Polym..

[32] Hiramoto K., Yamate Y., Sugiyama D., Matsuda K., Iizuka Y., Yamaguchi T. (2019). Effect of tranexamic acid in improving the lifespan of naturally aging mice. Inflammopharmacology.

[33] Sağlam M.F., Pulathan Z., Karahan S.C., Yuluğ E. (2024). Reduction in lung injury in an experimental ischemia-reperfusion model with tranexamic acid: A biochemical and histopathological assessment. Cardiovasc. Surg. Interv..

[34] Prudovsky I., Carter D., Kacer D., Palmeri M., Soul T., Kumpel C., Pyburn K., Barrett K., DeMambro V., Alexandrov I. (2019). Tranexamic acid suppresses the release of mitochondrial DNA, protects the endothelial monolayer and enhances oxidative phosphorylation. J. Cell Physiol..

[35] Brochard S., Boumédiene K., Mercier J., Agin V., Conrozier T., Baugé C. (2024). A single intraarticular injection of a tranexamic acid-modified hyaluronic acid (HA-TXA) alleviates pain and reduces OA development in a murine model of monosodium iodoacetate-induced osteoarthritis. Front. Pharmacol..

[36] Lin S., Zhang X., Xia X., Xu G., Pan H. (2025). Study on the Effects of Tranexamic Acid on Perioperative Inflammation, Long-Term Functional Recovery, and Satisfaction in Total Knee Arthroplasty: A Follow-up Study. J. Inflamm. Res..

[37] Mukasa F., Baba T., Hayashi K., Watari T., Ishijima M. (2025). Effects of preoperative systemic administration of tranexamic acid alone on postoperative inflammation and pain in total hip arthroplasty: A retrospective cohort study. Arthroplasty.

[38] Rui C., Dai G., Tian C., Zhou S., Gao Y., Cao M., Wu W., Qin S., Rui Y. (2025). Anti-inflammatory effect of multi-dose tranexamic acid in hip and knee arthroplasty: A systematic review and meta-analysis of randomized controlled trials. Inflammopharmacology.

